# Prenatal Renal Vein Thrombosis

**DOI:** 10.3390/children12030319

**Published:** 2025-02-28

**Authors:** Petya Markova, Ivanka Paskaleva, Stoyan Markov, Mariya Neshterova

**Affiliations:** 1 Department of Pediatrics, Medical University of Plovdiv, 4000 Plovdiv, Bulgaria; petya.markova@mu-plovdiv.bg (P.M.); ivanka.paskaleva@mu-plovdiv.bg (I.P.); 2 Department of Pediatrics, University Hospital “St. George”, 4000 Plovdiv, Bulgaria; 3 Department of Otorhinolaryngology, Medical University of Plovdiv, 4000 Plovdiv, Bulgaria; 4 Department of Otorhinolaryngology, University Hospital “St. George” Plovdiv, 4000 Plovdiv, Bulgaria; 5 Department of Neonatology, USHATOG Selena Plovdiv, 4000 Plovdiv, Bulgaria; neshterova.maria@gmail.com

**Keywords:** renal vein thrombosis, chronic kidney disease

## Abstract

**Background:** The neonatal period has a number of characteristics leading to an increased risk of severe and, in many cases, life-threatening complications. Renal venous thrombosis is one of them. It accounts for 16–20% of all thromboembolisms in the neonatal period. Due to the delicate balance in coagulation status in the first days after birth, conditions such as infections, hypoxia, hypotension, and dehydration can lead to the occurrence of this complication. The incidence of renal thrombosis is 2.2/100,000 live births, with cases of intrauterine renal thrombosis being even rarer (7% of cases). The diagnosis of the disease is usually performed using ultrasound examination and Doppler sonography, although contrast angiography is the gold standard for diagnosing these conditions. **Case presentation:** We present a clinical case of a male child with manifestations of diabetic fetopathy and prenatally occurring venous thrombosis of the right kidney, confirmed by ultrasound 2 h after birth. **Results:** The occurrence and evolution of venous thrombosis was monitored through a series of ultrasound examinations. Despite the restoration of renal blood flow after the initiation of therapy, long-term follow-up at 6 and 12 months revealed the onset of renal atrophy. **Conclusions:** Prenatal renal vein thrombosis is a rare but severe pathology for the newborns. Ultrasound examination is the method of first choice in cases of suspected renal vein thrombosis, as well as for renal blood flow restoration and for the monitoring of the fate of the affected kidney.

## 1. Introduction

The occurrence of thromboembolism in the intrauterine and neonatal periods is a serious complication that can lead to a fatal outcome. Rayer first described renal vein thrombosis in 1837 [1]. A significant contribution to the elucidation of the pathophysiology of this process was made by Hepler, who in 1934 published a paper describing how thrombosis begins in a small arcuate or interlobular renal vein and can spread in both directions to involve the renal cortex or occlude the main renal vein [2]. Campbell et al., in 1942, spoke of the surgical treatment of this condition (nephrectomy) as opposed to conservative treatment. However, in the following years, the surgical approach was abandoned in favor of conservative therapy, which gave much better results [2].

According to the statistics, renal vein thrombosis is more common than renal artery involvement [3]. Renal vein thrombosis accounts for 16–20% of all thromboembolisms in the neonatal period [4]. In 80% of cases, the onset of the disease is in the first 30 days after birth [5], most often in the first 1–3 to 7 days, but it can also be observed in the intrauterine period. According to the clinical data, the frequency is about 2.2/100,000 live births, and in premature babies it is six times higher. In about 30% of cases, the renal thrombosis spreads to the inferior vena cava, and in about 10% it is accompanied by adrenal hemorrhage [6]. Both sexes are affected, with a male predominance [4,7]. Unilateral renal thrombosis is much more common than bilateral, with a left kidney involvement prevalence [8]. Factors such as premature birth, hypotension, dehydration [9], diabetes, polycythemia, congenital heart disease, and indwelling umbilical venous catheters are predisposing factors for the development of this complication [6,7]. It usually begins gradually as an incomplete thrombosis of the main renal vein or its branches, gradually developing into a complete one.

## 2. Case Report

We present the clinical case of a male neonate, born at 34 weeks of gestation by urgent operative delivery to a mother with diabetes type I and preeclampsia. It was performed due to the absence of spontaneous fetal movements for approximately 6 h, as reported by the mother. His birth weight was 2550 g, his length was 45 cm, and there were data for perinatal asphyxia—with an Apgar score of 6 at the 1st min and 8 at the 5th min. After cardiopulmonary resuscitation in the delivery room, the child was admitted to the intensive neonatal care unit (NICU) and placed on nasal continuous positive airway pressure (nCPAP).

Clinical examination revealed morphological signs of diabetic fetopathy.

Respiratory system—tachypnea, dyspnea, and weakened bilateral vesicular breathing with crepitations.

Cardiovascular system—rhythmic normofrequent heart rate, clear tones, and good peripheral arterial pulsations bilaterally.

Abdomen—above the chest level, distended, with a palpable dense structure along the entire right abdominal site.

Neurological status—generalized muscular hypotonia and hyporeflexia. Head—properly configured, anterior fontanelle size 10/10 mm, at the level of the skull bones, posterior fontanelle—opened, cranial sutures partially fused. Male genitalia.

The blood tests conducted on admission and their dynamics are shown in Table 1.

**Figure 1:**
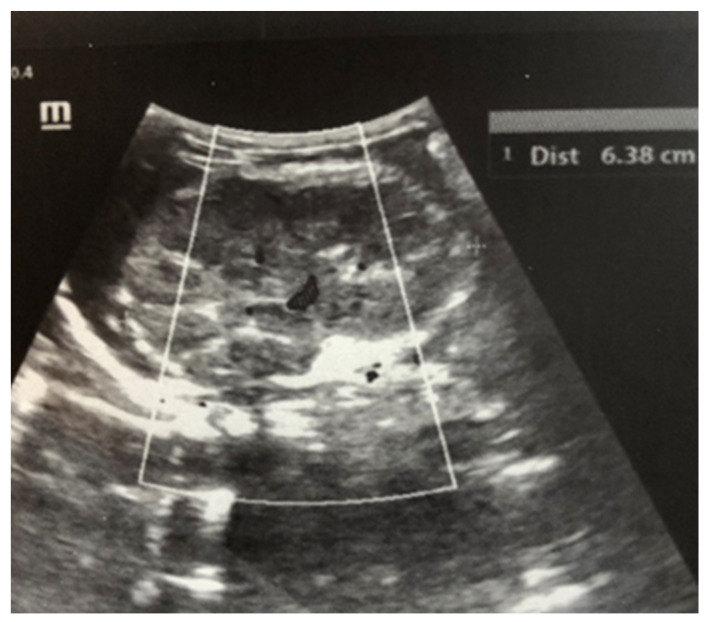
Right kidney image taken in the 2nd hour after birth. There is an increase in size, increased echogenicity of the parenchyma, and interlobar hyperechoic spikes.

**Figure 2:**
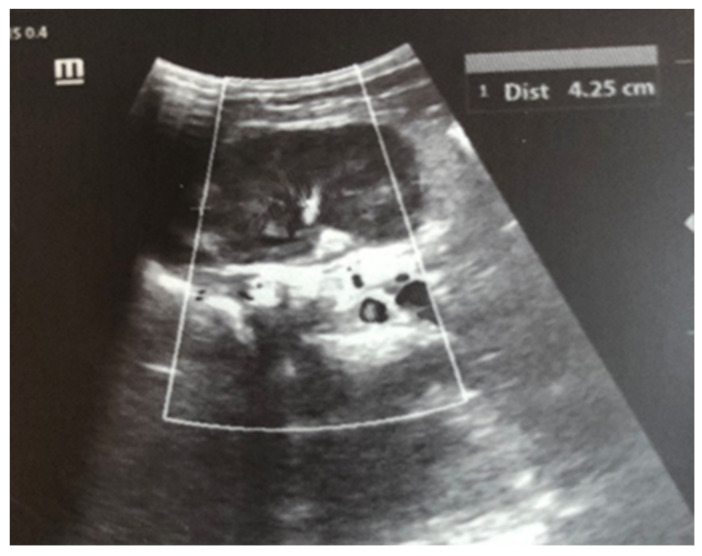
Left kidney image taken in the 2nd hour after birth—the topic, size, and echogenicity of the parenchyma are preserved, without drainage disorders.

After admission to the NICU, the neonate was found febrile, with symptoms of multiple organ failure: respiratory system failure requiring treatment with Curosurf and mechanical ventilation; renal failure with symptoms of oliguria, macroscopic hematuria, and an increase in nitrogen waste products; the manifestation of neurological symptoms—muscular hypertonia and hyperreflexia, increased convulsive readiness with frequent spontaneous and provoked clonuses of the limbs. The blood tests revealed inflammatory activity—increased CRP and evidence of early nonconjugated hyperbilirubinemia. An abdominal ultrasound examination, performed 2 h after birth, found an enlarged right kidney with a longitudinal size of 6.1 cm, swollen parenchyma with a thickness of 1.8 cm, and increased echogenicity with the presence of interlobar hyperechogenic spikes—an image characteristic of the early phase of renal vein thrombosis. The left kidney—with a longitudinal size of 4.1 cm—preserved the topic, size, and echogenicity of the parenchyma, without drainage disorders, see Figure 1 and Figure 2.

**Figure 3:**
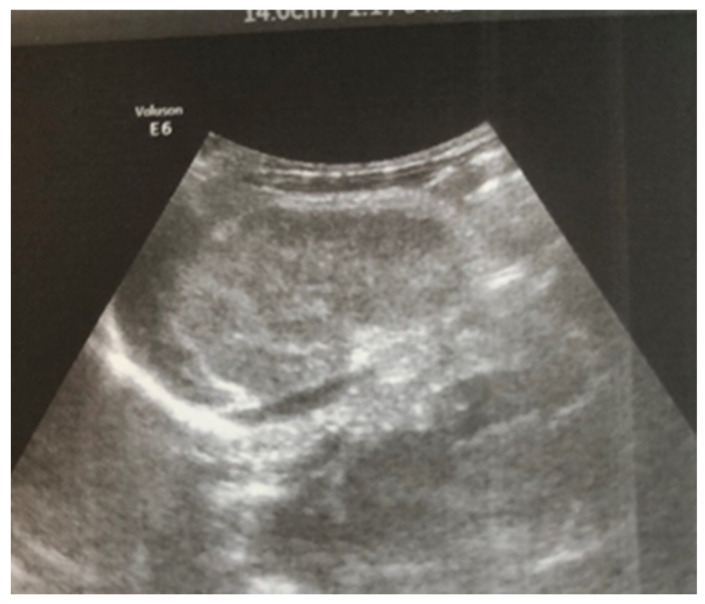
Right kidney image taken on the third day after birth—increased size, increased echogenicity of the parenchyma, and loss of cortico-medullary differentiation.

**Figure 4:**
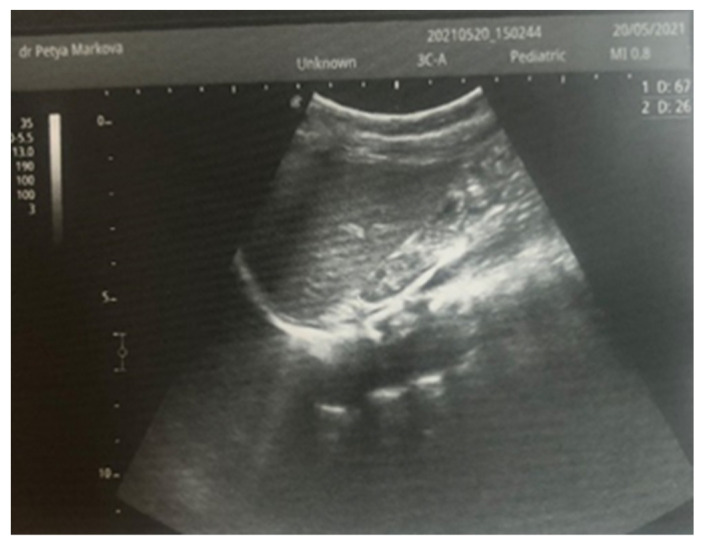
Right kidney image at 1 year of age—reduced in size, with a longitudinal size of 2.6 cm, increased echogenicity of the parenchyma, and the presence of a small subcortical cyst.

Complex therapy was performed—mechanical ventilation, antibiotics in doses adjusted according to creatinine clearance, anticonvulsants, low-molecular-weight herapin (Enoxaparin), inotropic (Dopamine), and phototherapy. The child’s condition gradually improved and he was extubated on the 9th day of the stay in the NICU. An uncomplicated post-extubation period followed. In the following 2–3 days, macroscopic hematuria was observed. On the second day after extubation, the child’s diuresis was good. The condition of the right kidney was monitored by ultrasound on the 3rd day (Figure 3), the 10th day (recovery of renal venous blood flow was observed), and at the end of the first month. On the 10th day, a decrease in kidney size was noted, and at the end of the first month, the size was below the norm for the age—i.e., the onset of renal atrophy. Follow-up examinations at 6 months and 1 year showed evidence of the atrophying of the right kidney with a longitudinal size of 2 cm, the occurrence of compensatory hypertrophy of the left kidney, normal blood pressure, and preserved renal function (Figure 4).

## 3. Discussion

Thromboembolic complications of the renal blood vessels are rare but serious and sometimes potentially fatal in children. The renal vein is much more commonly affected than the renal artery. Renal vein thrombosis most commonly occurs in the neonatal period [3]. The risk factors for its occurrence can be divided into two main groups: maternal risk factors and fetal risk factors [3] (Table 2). In our case, risk factors were identified on both the mother’s side—preeclampsia and diabetes—and the fetus’s side—perinatal asphyxia.

Males are more often affected—our patient is a male too. Most cases have clinical manifestation within the first three days after birth (67%). In 7% of cases, renal thrombosis can occur intrauterinely. Given the presence of clinical findings detected immediately after birth—abdominal asymmetry with a palpable dense structure along the entire right abdominal side—we consider the venous thrombosis, diagnosed by us in the 2nd hour after birth, to have occurred prenatally. The classic clinical presentation, including the triad of a palpable mass in the left or right flank, a macroscopic hematuria, and thrombocytopenia (as observed in our case as well) occurs in about 22% of cases. In about 25% of cases, thrombosis may be bilateral. It can also be asymptomatic and discovered during an ultrasound examination performed for another reason or it can manifest with the clinical signs of acute kidney injury.

## 4. Imaging Studies

Ultrasound examination is the main tool for the detection and follow-up of renal vein thrombosis [10]. Neonatal venous thrombosis usually arises gradually from the small venous branches and spreads to the main renal vein. In the first stage of thrombosis, interlobular and interlobar hyperechoic stripes are observed, the kidney is enlarged in size, and it becomes spherical with hypoechoic pyramids [7]. These hyperechoic stripes are in fact the thrombosis of the small interlobular and/or interlobar veins with perivascular hemorrhage and edema [11]. In the later stage, after the first few days, the kidney becomes heterogeneous, with a loss of cortico-medullary differentiation. Doppler ultrasound examination may show reduced amplitude or a missing venous signal. After about two weeks, a decrease in the size of the injured kidney occurs, along with hypoechogenicity in the area of the medulla, caused by the liquefaction (liquefaction) of the kidney tissue. Over time, in a large percentage of cases, atrophy develops with the renal function decrease and contralateral hypertrophy of the other kidney [5,7]. In most cases, ultrasound is sufficient for diagnosis, but in unclear cases, a CT scan of the kidneys may be performed. In the presented case, the diagnosis and disease progression were also monitored through ultrasound surveillance, performed daily during the first days after birth. This allowed for the tracking of the characteristic changes in renal venous thrombosis in the neonatal period. Due to the critical condition of the infant, contrast angiography was not performed.

## 5. Treatment

There is no clear protocol for renal thrombosis treatment. Therapeutic options include maintenance therapy, anticoagulants, and thrombolysis. The maintenance of the water–electrolyte balance, the alkaline–acidic state, and nutrition in all newborns with renal thrombosis is extremely important. The choice of therapy must consider the potential benefits of anticoagulants and thrombolytic treatment and the risk of intracranial hemorrhage with all its consequences. In cases of spontaneous unilateral thrombosis, without evidence of acute renal injury (AKI) and the spread of the thrombosis to the inferior vena cava, the use of unfractionated or low-molecular-weight heparin in therapeutic doses is appropriate, with the strict monitoring of prothrombin time and anti-factor Xa levels. In cases of unilateral thrombosis and AKI, or unilateral thrombosis without AKI but with spread to the inferior vena cava, unfractionated heparin therapy is also performed and/or is subsequently replaced with low-molecular-weight heparin until renal venous blood flow is restored for a total period from 6 weeks to 3 months. In cases of bilateral renal venous thrombosis, thrombolytic treatment with tissue plasminogen activator is initiated, followed by unfractionated heparin or low-molecular-weight heparin [2,4]. According to Messinger Y. et al., in a large percentage of cases, regardless of whether anticoagulant therapy or only supportive therapy is administrated, renal atrophy eventually occurs [8]. After the first 3 months, it is also advisable to administer dimercaptosuccinic acid (DMSA). Scintigraphy is to be performed to determine the percentage of function of the affected kidney. Late consequences after renal venous thrombosis include renal atrophy, chronic kidney disease, or hypertension. In our case, we chose therapy with low-molecular-weight heparin—Enoxaparin, because the thrombosis affected only the right kidney, without extending to the vena cava and without evidence of the involvement of the contralateral kidney.

The therapy we initiated was timely in relation to the moment of diagnosis but not in terms of the onset of the disease, as this thrombosis occurred prenatally. Its prenatal origin is supported by the ultrasound image we observed immediately after birth, along with its dynamic changes, which are characteristic of the first three days of this type of thrombosis.

Although renal blood flow was restored by the 10th day, progressive atrophy of the affected kidney gradually occurred in the case presented (Figure 4). According to a study by Winyard et al. involving 23 children with neonatal thrombosis over a 15-year period [12], the length of the affected kidney exceeding 6 cm (as in our case) is typically associated with the development of renal atrophy, regardless of the treatment administered.

## 6. Conclusions

The presented clinical case illustrates the rare (7%) prenatal renal venous thrombosis in a case of a newborn with fetal risk factor: perinatal asphyxia and the maternal risk factors type 1 diabetes and preeclampsia. Due to the severe condition of the child and his placement on mechanical ventilation, CT angiography could not be performed, but with a series of ultrasound examinations, we diagnosed and monitored the dynamics of typical ultrasound changes observed in neonatal renal venous thrombosis.

Despite the initiation of treatment with low-molecular-weight heparin, renal atrophy of the affected kidney occurred. A similar outcome is described in most cases with neonatal thrombosis, regardless of the treatment, especially when the size of the kidney is over 6 cm, as it was in our case. Ultrasound examination is the method of first choice in cases of suspected renal vein thrombosis, as well as for renal blood flow restoration and for the monitoring of the fate of the affected kidney. 

## Figures and Tables

**Table 1:** Blood tests on admission and dynamics.

Parameter	16.11.20	18.11.20	26.11.20	03.12.20
Hb	157 g/L	150 g/L	147 g/L	130 g/L
RBC	4.71 × 10/12/L	4.62 × 10/12/L	4.83 × 10/12/L	4.27 × 10/12/L
WBC	59.9 × 10/9/L	75.7 × 10/9/L	11.3 × 10/9/L	12.3 × 10/9/L
PLT	178 × 10/9/L	212 × 10/9/L	475 × 10/9/L	468 × 10/9/L
CRP	57.6 mg/L	29 mg/L	2 mg/L	1.6 mg/L
Creatinine	173 µmol/L	167 µmol/L	60 µmol/L	45 µmol/L
Urea	9.8 mmol/L	18.7 mmol/L	13 mmol/L	8.9 mmol/L
LDH	5705 IU/L	4211 IU/L	1353 IU/L	710 IU/L
Bilirubin	327 µmol/L	428 µmol/L	48.8 µmol/L	
Direct bilirubin	14 µmol/L	18 µmol/L	9 µmol/L	
pH	7.1	7.55		
BE	−5.6	−2.5		
HCO_3_^−^	19.6	26.3		
Urine				
Protein	3+	4+	1+	-
Blood	+	+	-	-
WBC	-	-	-	-
Sediment	28–30 RBC, 1–2 WBC	Abundance of erythrocytes	1–2 RBC	1–2 WBC

**Table 2:** Renal vein thrombosis risk factors.

Neonatal Thrombosis	
Neonatal/fetal factors	Maternal factors
Catetherizacions Congenital heart disease Respiratory distress syndrome Sepsis Dehydration Perinatal asphyxia Polycythemia Congenital thrombophilia Prematurity	Infertility Oligohydramnios Thrombophilia Chorioamnionitis Preeclampsia Diabetes Autoimmunity/anti-phospholipid syndrome/ Intrauterine death of twins

## Data Availability

Data available on request from the authors.
